# Strain-induced Mn valence state variation in CaMnO_3−*δ*_/substrate interfaces: electronic reconstruction *versus* oxygen vacancies

**DOI:** 10.1039/d3na00206c

**Published:** 2023-07-04

**Authors:** Van-Hien Hoang, Nam-Suk Lee, Heon-Jung Kim

**Affiliations:** a Department of Physics, Graduate School, Daegu University Gyeongbuk 38453 Republic of Korea; b National Institute for Nanomaterials Technology (NINT), Pohang University of Science and Technology (POSTECH) Pohang 37673 Republic of Korea nslee@postech.ac.kr; c Department of Materials-Energy Science and Engineering, College of Engineering, Daegu University Gyeongbuk 38453 Republic of Korea hjkim76@daegu.ac.kr

## Abstract

This study investigates the nanoscale crystalline and electronic structures of the interfaces between CaMnO_3−*δ*_ and substrates such as SrTiO_3_ (001) and LaAlO_3_ (001) by employing advanced transmission electron microscopy and electron energy loss spectroscopy techniques. The objective is to comprehend the influence of different strains on the Mn valence state. Our findings reveal that the Mn valence state remains relatively stable in the region of a weakly tensile-strained interface, whereas it experiences a significant decrease from Mn^4+^ to Mn^2.3+^ in the region of a strongly tensile-strained interface. Although this reduction in valence appears to be consistent with the electron reconstruction scenario, the observed increase in the out-of-plane lattice constant at the interface implies the accumulation of oxygen vacancies at the interface. Consequently, the present study offers a comprehensive understanding of the intricate relationships among the Mn valence state, local structure, and formation of oxygen vacancies in the context of two distinct strain cases. This knowledge is essential for tailoring the interface properties and guiding future developments in the field of oxide heterostructures.

## Abstract 2

The Mn valence state near the interfacial region between the bottom CMO layer and substrate varies when the bottom CMO layer experiences different levels of tensile strain induced by the substrates.

## Graphical abstract

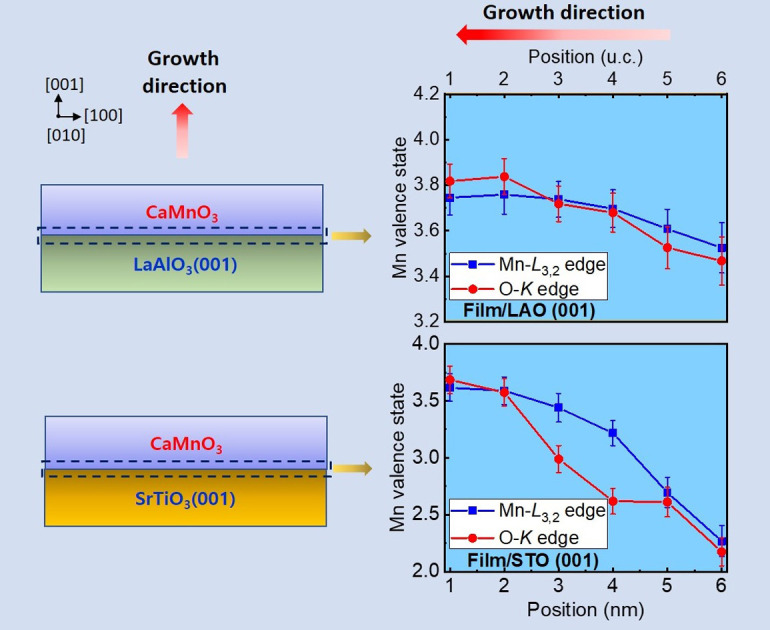

## Introduction

Perovskite oxides (ABO_3_) are a diverse group of materials that showcase a range of unique properties, such as high-temperature superconductivity, colossal magnetoresistance, and various magnetic and ferroelectric orders.^1,2^ La_0.7_Ca_0.3_MnO_3_ (LCMO) and related compounds are of great interest due to their interplay between lattice, spin, and charge degrees of freedom, resulting in half-metallicity, ferromagnetism at room temperature, and colossal magnetoresistance, making it an ideal platform for various applications. While bulk LCMO is a metallic ferromagnet, ultrathin LCMO films become insulators with reduced magnetization below a critical thickness of 2 nm (or 4 unit-cells).^3^ This phenomenon referred to as a “dead layer”, is where magnetic and metallic properties are suppressed and remains largely a mystery despite extensive investigation. While some researchers attribute the dead layer to structural disorder or chemical modification,^4,5^ others suggest it could be due to oxygen coordination, interfacial oxygen octahedral rotation, interfacial strain, and oxygen vacancy concentration.^6–9^ To gain a deeper understanding of the dead layer's origin, researchers are using interface-selective probing techniques like magnetization-induced second-harmonic generation^10^ and high-resolution transmission electron microscopy (HRTEM).^11–14^ However, the interplay between structural strain, oxygen stoichiometry, and chemical composition remains poorly understood. In this study, we address this issue.

Heterostructures are frequently used in various applications where interfaces play a critical role in determining their properties. Several factors such as strain, charge transfer, and spin-exchange interactions can significantly impact the magnetic and electronic properties of interfaces.^15–17^ It is crucial to carefully control the interfaces between two materials, as they can exhibit new physical properties that are not present in the individual materials. In this case, electrostatic boundary conditions can play a crucial role in controlling the atomic and electronic structures at the interface. For instance, the polar discontinuity across the interface can cause severe roughening or significant reconstruction as a means of avoiding the diverging electrostatic potential.^18,19^ In perovskite oxides containing a transition metal, mixed-valence states allow for electronic reconstruction at the interface, which helps overcome the polar discontinuity. This redistribution of electrons at the interface leads to the suppression of the electrostatic potential.^19^

The accumulation of charged defects or impurities, such as oxygen vacancies, can also circumvent the polar discontinuity at the interface. This leads to an interface electronic structure that deviates from the bulk structure. This may result in significant consequences, as enhanced oxygen vacancies near the interface can play a crucial role. For instance, the concentration of oxygen vacancies in heterostructures has been linked to significant changes in electrical resistance, which leads to a metal-insulator transition.^20^ The presence of a conducting electron layer between two bulk insulators, such as STO and LAO, has gained considerable attention. Although initially attributed to polar discontinuities leading to the formation of an electron gas at the interface,^21^ recent studies suggest that electron doping is mainly due to oxygen vacancies in the STO layer during growth.^22,23^ Furthermore, oxygen vacancies near interfaces are believed to be responsible for magnetism found in non-magnetic heterostructures^24,25^ and are thought to contribute to their catalytic activity.^26,27^ These findings highlight the importance of manipulating oxygen vacancies in determining the emergent properties of heterostructures, and further control of oxygen vacancy concentration and distribution are necessary. To fully understand and apply oxide heterostructures, it is crucial to study the relationship between oxygen vacancies and the valence state of the B-site cation at the interface. However, this is a challenging task due to the small length scale and complex structure and chemistry at interfaces. So far, only a few measurements have provided direct information about the valence state distribution at the interface.

We studied CaMnO_3−*δ*_ (CMO) thin films grown on (001)-oriented LAO and STO substrates using pulsed laser deposition (PLD) to gain insight into the interface between the film and substrate. The substrates were selected to impose different strain effects on the films; the STO substrate imposed strong tensile stress, while the LAO substrate imposed weak tensile stress. In the past, several experimental and theoretical studies have been conducted on tensile strain-induced interfacial oxygen vacancies. For example, Ulrich Aschauer *et al.* have investigated the stability of biaxially strained *Pnma* perovskites CMO towards oxygen vacancies using first-principle calculations. They found that the energetics of oxygen vacancy formation in biaxially strained CMO films with *Pnma* structure are strongly influenced by strain, with tensile strain favoring oxygen vacancy formation.^28^ However, no studies comparing the accumulation of oxygen vacancies at the interface between films grown on different substrates have been conducted.

The method involves analysing the core-loss in the characteristic L edge of transition metals, which is caused by the excitation of two *p*-levels to three largely unoccupied levels.^29^ The relative intensities, shapes, and threshold energies of the L_2_ and L_3_ peaks are used to quantify the number of d-electrons.^30^ Additionally, changes in the O-K near-edge fine structure can also indicate modifications in the *d*-oxidation state, which can be used to obtain information on the d-band occupancy (*i.e.*, valence or oxidation states).^31,32^ As a result, core-loss EELS can be utilized to determine the valence state of transition metals.

## Experimental section

In this study, two tri-layers of CMO/LCMO/CMO were grown on LAO (001) and STO (001) substrates using a pulsed laser deposition (PLD) technique. The top and bottom CMO layers had a fixed thickness of 4 nm in both samples, while the middle LCMO layers had thicknesses of 30 nm on LAO (film A) and 4 nm on STO (film B). A comparison was made with a bilayer of LCMO/CMO/STO (001) sample (film C), which was grown under the same conditions as the above two samples. The thicknesses of CMO and LCMO in this sample were 4 nm and 30 nm, respectively. In their bulk forms, CMO and LCMO have lattice parameters of 3.72 Å and 3.86 Å, respectively. The films were expected to experience weak tensile strain along the in-plane of the LAO substrate (*a* = 3.79 Å), while strong tensile strain was expected through the STO substrate (*a* = 3.905 Å). The growth temperature was 700 °C and the partial oxygen pressure was 10^−1^ torr, with a laser fluence of 1.0 J cm^−2^ and a laser frequency of 2 Hz. After deposition, the samples were cooled down to room temperature at an oxygen pressure of 500 torr. The CMO and LCMO polycrystalline targets of high quality were synthesized using the solid-state reaction presented elsewhere.^33^ The samples were characterized for their crystallinity using X-ray diffraction with synchrotron radiation at the 3A beamline of the Pohang Light Source (PLS) in South Korea. We mainly investigated the film/substrate interface.

The thin films were analyzed using cross-sectional scanning transmission electron microscopy (STEM) and electron energy loss spectroscopy (EELS). Samples were cut along the [100] direction of both LAO and STO and prepared for STEM examination through mechanical grinding, polishing, and dimpling, followed by Ar-ion milling at 4.5 kV. Energy-loss spectra were acquired on a Philips CM200 FEG electron microscope operating at 200 kV. Elemental mapping for elements La, Ca, Sr, Al, Mn, and Ti was performed using a multiple linear least square fitting method. Energy-loss near-edge structure analysis was performed with a 0.5 eV per channel dispersion and an energy resolution of 2.3 eV, as measured by the full-width at half-maximum of the zero-loss peak.

## Results and discussion

**Fig. 1:**
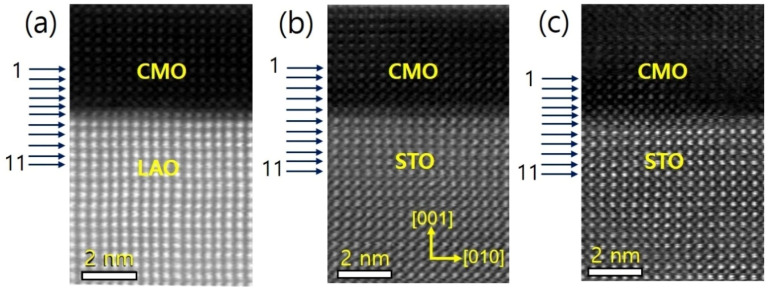
The HAADF-STEM images of films A (a), B (b), and C (c) were taken in the cross-sectional direction along the [100] zone axis, and the 11 positions at which EELS were measured are marked by horizontal arrows. The images show the film/substrate interfaces. The bottom CMO layer has a thickness of approximately 4 nm.

The sample structure was analyzed using X-ray diffraction, as described in the previous report.^34^ The thin films were further examined using STEM, and the resulting images are presented in Fig. 1(a)–(c) for films A, B, and C, respectively. The EELS measurements were taken at 11 positions indicated by horizontal arrows. The images reveal a sharp atomic-scale interface between the bottom CMO layers and substrates, with the CMO layers appearing darker and the substrates appearing brighter. All atomic columns are resolved in the images, except for O columns, which exhibit weak electron scattering. The lattice fringes extend across the interface from the substrate to the film, with no visible defects or cation intermixing.

**Fig. 2:**
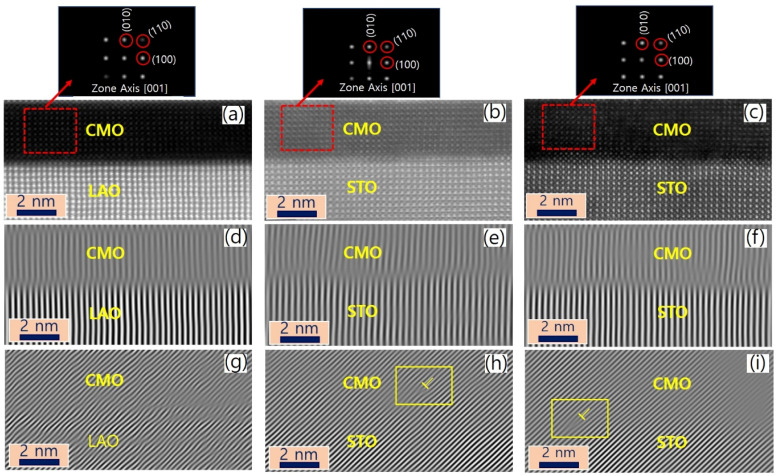
The interface between the films and substrates was analyzed using HAADF-STEM and FFT images. (a)–(c) HAADF-STEM images of films A, B, and C near the interface between the film and substrate, respectively. The areas marked with red dashed squares in the corresponding STEM images are shown in FFT images on the top. (d)–(f) and (g)–(i) Inverse FFT lattice fringe images, projected in the [001] and [110] directions, corresponding to (a), (b), and (c), respectively. The misfit dislocation core near the film/substrate interface is indicated by the yellow dashed region.

Cross-sectional images in the vicinity of interfaces of films A, B, and C are presented in Fig. 2(a)–(c), viewed along the [001] zone axis. Large views of STEM and energy-dispersive X-ray spectroscopy (EDS) images to confirm the uniformity of the heterostructures were presented in the previous report.^34^ The fast Fourier transform (FFT) images of the regions denoted by red dashed boxes in the corresponding STEM are displayed on top of Fig. 2(a)–(c). The reflection peaks from (001), (110), and (100) planes, which constitute a pseucubic structure, confirm that the octahedra are not rotated or tilted along the growth direction. To further clarify the epitaxial relationship, we filtered the STEM images using inverse Fourier transform projected along the [001] and [110] directions, as shown in Fig. 2(d)–(f) and (g)–(i), respectively. For film A, no misfit dislocation core is observed in either projection direction [Fig. 2(d) and (g)]. In contrast, for films B and C, misfit dislocation cores are detected in the [110] projection near the interface between the film and the substrate, as marked by the yellow dashed box [Fig. 2(h) and (i)]. This result agrees with the reciprocal space mapping (RSM) measurement in the previous report,^34^ where additional broadening of the film spot was observed in the RSM measurement due to the presence of misfit dislocations.^35^ These findings suggest the existence of misfit dislocations in the strongly tensile-strained interface on the STO substrate, as revealed by the STEM images.

**Fig. 3:**
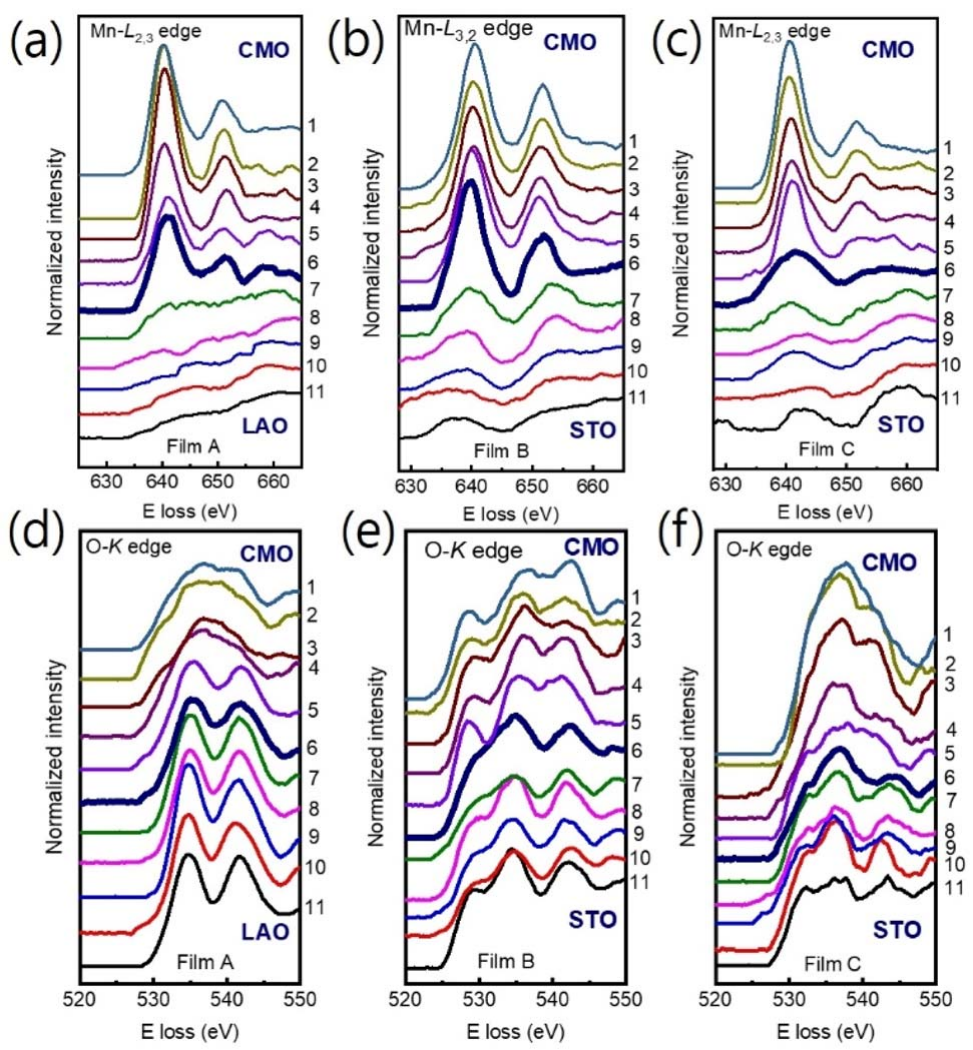
(a)–(c) The Mn L_3,2_ and O-K edges (d)–(f) position-dependent EELS spectra were obtained across the film/substrate interface for films A, B, and C, respectively. Spectra were extracted from positions 1 to 11, as indicated in Fig. 1, with the bolder spectrum indicating the interface position. For clarity, the spectra were vertically displaced.

We used Mn-L_3,2_ and O-K core loss edges to explore the electronic structures of the film/substrate interfaces. Fig. 3(a)–(c) show Mn-L_3,2_ edges for 11 different positions across the interfaces, marked as horizontal arrows in Fig. 1(a)–(c) for films A, B, and C, respectively. Spectra 1–5 were acquired from the films, spectra 7–11 from the LAO (STO) substrate, and spectrum 6 from the interfacial position (bolder marked lines). The Mn-L edges consist of two peaks, L_3_ and L_2_ lines,^31,32,36,37^ which were found around 641 eV and 651 eV, respectively. Fig. 3(d)–(f) display the O-K edges for films A, B, and C, respectively. The O-K EELS shows three main peaks: a pre-peak positioned at ∼528 eV, a second peak around ∼535 eV, and the third peak at about ∼541 eV.^13,31,32^

**Fig. 4:**
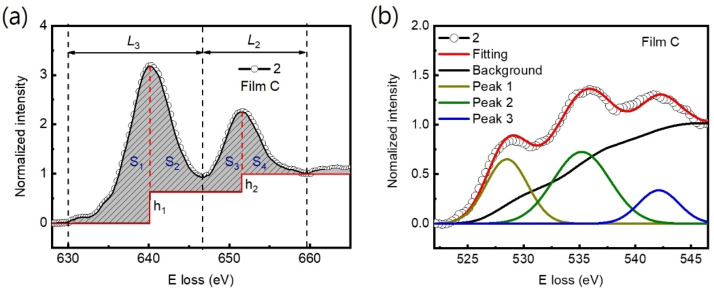
(a) Graph illustrating a generic Mn L_3,2_ spectrum, as well as the approximate location of the windows integrating L_3_ and L_2_ line. This is also the window used for scaling the step function. After scalling and substraction of this function, all remaining signals under the dashed L_3_ (S_1_ + S_2_) and L_2_ (S_3_ + S_4_) lines are integrated, and their ratios are calculated. (b) O-K edges were additionally employed to estimate the Mn valence state. The normalized pre-peak intensity is represented by the relative area under the fitting curves for peak 1 (solid olive) and peak 2 (solid green).

**Fig. 5:**
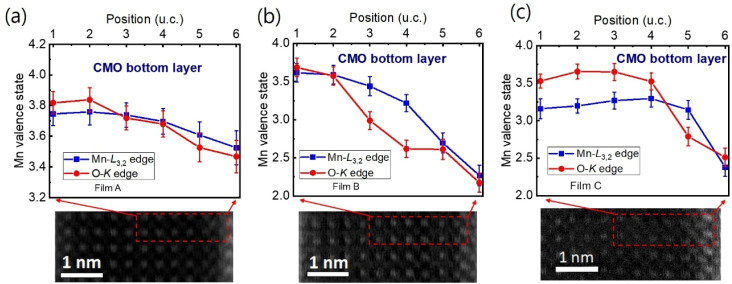
(a)–(c) The Mn valence state varies with the position near the film/substrate interfaces for films A, B, and C, respectively. The valence state was estimated using two methods: Mn L_3_/L_2_ white-line ratio [blue symbols and lines] and O-K edge [red symbols and lines]. The positions where the valence state was measured are indicated by red dashed boxes in the bottom STEM images.

The Mn valence states were estimated from the Mn L_3_/L_2_ white-line intensity ratios, as detailed in Fig. 4(a).^31,38,39^ After removing the background below the Mn L_3,2_ edge by using a power law fit, we scale a step function and subtract it from the Mn L_3,2_ edge to remove the continuum contribution. A 13 eV wide window placed right after the L_2_ line is used for scaling. These windows are the first at the onset of the L_3_ line and the second next to it. Based on the multiplicity of the initial states (four 2p^3/2^ electrons and two 2p^1/2^ electrons), the ratio of the step heights *h*_1_ and *h*_2_ is chosen to be 2 : 1.^40^ In the next step, the remaining signal under the corrected L_3_ (S_1_ + S_2_) and L_2_ (S_3_ + S_4_) lines was integrated within two 13 eV wide windows. Using the integrated intensity values, the L_3,2_ intensity ratio was calculated.^38^ From the L_3,2_ ratio, Mn valence was estimated.^31^ The obtained Mn valence states for films A, B, and C are shown as blue solid squares and lines in Fig. 5(a)–(c), respectively, revealing a clear trend of decreasing Mn valence state from the bottom CMO layer towards the substrate interface. This trend is consistent with previous reports on La_*x*_Sr_1−*x*_MnO_3_, (La, Ca)MnO_3_ and TbMnO_3_ grown on STO substrates.^13,41–43^ However, the reduction in Mn valence near the film/substrate interface for films B and C (∼+2.3) is much larger than that for film A (∼+3.5).

The Mn valence state is also estimated based on O-K edges. The method for estimating is presented in Fig. 4(b).^31,32,44^ First, a power-law function was applied to subtract the background. Then fitting of the normalized O-K spectra was carried out by means of a Shirley background and Lorentzian–Gaussian functions combined. Three components were deconvoluted from the resultant spectra. Peak 1 of the O-K EELS results from the excitation of O 1s core electrons into unoccupied O p orbitals hybridized with Mn 3d orbitals, which are sensitive to the occupancy of the 3d band. In peak 2, unoccupied O 2p orbitals are hybridized with Ca 4d orbitals, and in peak 3, Ca 4sp orbitals are hybridized with Mn 4sp orbitals.^11,31,32^ Lastly, normalized pre-peak intensity is represented by the relative area under the fitting curves for peak 1 (solid olive) and peak 2 (solid green). Based on the normalized pre-peak intensity value, the Mn valence was estimated.^31^ As expected, the Mn valence states obtained from the O-K edge (red circles and lines in Fig. 5(a)–(c)) in the vicinity of the film/substrate interfaces show a similar trend with a dependence on a position as that from the Mn-L_3,2_ edge.

The distribution of Mn valence states at the film/substrate interface is found to depend on the substrate, with a significant reduction occurring near the strongly tensile-strained interface. As CMO and STO are nonpolar along the [001] direction, electronic reconstruction is unlikely in the CMO/STO interface, making the sharp decrease in the Mn valence state likely due to the accumulation of oxygen vacancies at the interface. Several previous studies have also reported the presence of oxygen vacancies at the film/substrate interface.^13,14,41^ Additionally, the accumulation of oxygen vacancies in the strongly tensile strained CMO films using first-principle calculations have been reported.^28^

The Mn valence state reduction observed at the film/substrate interface in tensile-strained films could also be affected by misfit dislocations. The misfit dislocations may result in higher oxygen defect concentration at the interface than in the bulk due to a decrease in oxygen vacancy formation energy in the dislocation core.^45^ This is consistent with the inverse FFT images shown in Fig. 2(h) and (i), where the misfit dislocation cores can be observed near the interface between films and substrates. Another possibility for the sharp reduction in the Mn valence state at the interface could be the formation of a second phase due to cation non-stoichiometry. For instance, an epitaxial thin layer of Ruddlesden-Popper (La_2_Sr_2_Mn_3_O_10_) structure with *n* = 3 has been achieved by sequential deposition of (La_1−*x*_Sr_*x*_MnO_3_)_*n*_ and SrO layers using the PLD technique.^46^ However, no such interface phase was observed in the pseucubic structure of the CMO layer of films B and C as observed in Fig. 2.

**Fig. 6:**
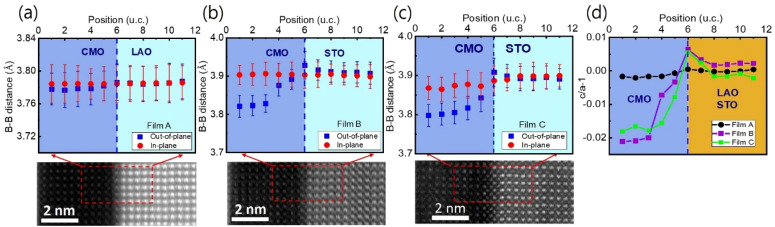
Local lattice parameters near the film/substrate interfaces: (a)–(c) display the local *c*-axis (blue solid square) and in-plane lattice (red solid circles) constants of films A, B, and C, respectively, close to the interface between the film and substrate. The 2D Gaussian function fitting was employed to estimate the local lattice constants from the HAADF-STEM images, with respect to the substrate (reference). The red dashed boxes in the HAADF-STEM images indicate the atomic positions across the interface where the local lattice constants were determined. Error bars represent the standard error for each lattice layer. (d) The (*c*/*a*) − 1 values near the film/substrate interfaces for films A (black solid circles and lines), B (violet solid circles and lines), and C (green solid squares and lines) were calculated as a function of position. Vertical blue dashed lines mark the interface position.

To investigate the local structure at the film/substrate interface with atomic resolution, we used a 2D Gaussian function to fit the atomic B–B columns from the ADF-STEM images, and calculated the local lattice constants.^47^ Here B represents the B-site ion, Mn in the present case. We measured the lattice constant relative to that of the substrate as a reference, along the out-of-plane and in-plane directions. Fig. 6(a)–(c) show the in-plane (solid red circles) and out-of-plane (solid blue rectangles) lattice constants as a function of atomic position at the interfacial region for films A, B, and C, respectively. The in-plane lattice distances of the bottom CMO layer are similar to those of the substrates and the values calculated from XRD data for all films.^34^ However, while the out-of-plane lattice constant of film A decreases slightly from the LAO substrate to the bottom CMO layer, films B and C show a slight increase in the out-of-plane lattice constant from the STO substrate to the interface, then a sharp decrease from the interface towards the bottom CMO layer. Note that the out-of-plane lattice parameter of the bottom CMO layer, as measured by STEM image, is smaller than the value obtained from the XRD experiment. This difference occurs because the out-of-plane lattice constant extracted from X-ray represents an average of both LCMO and CMO layers.

Fig. 6(d) provides a clearer view of how the local lattice constant behaves when considering the *c*/*a* − 1 value, which assesses the degree of tetragonality of each unit cell. It is noteworthy that while the *c*/*a* − 1 value remains nearly constant across the interface in film A, films B and C exhibit the maximum value at the interface (position 6). The Poisson effect predicts that the interfacial lattice in the out-of-plane direction should be compressed due to the stretching of the in-plane lattice. However, Fig. 6(b) and (c) do not show this behavior exactly at the interface. Instead, the unit cell precisely at the interface expands along the out-of-plane direction. The out-of-plane lattice expansion at the interface is not solely due to stress and also related to the reduction of the Mn valence for the strongly tensile-strained interface.

**Fig. 7:**
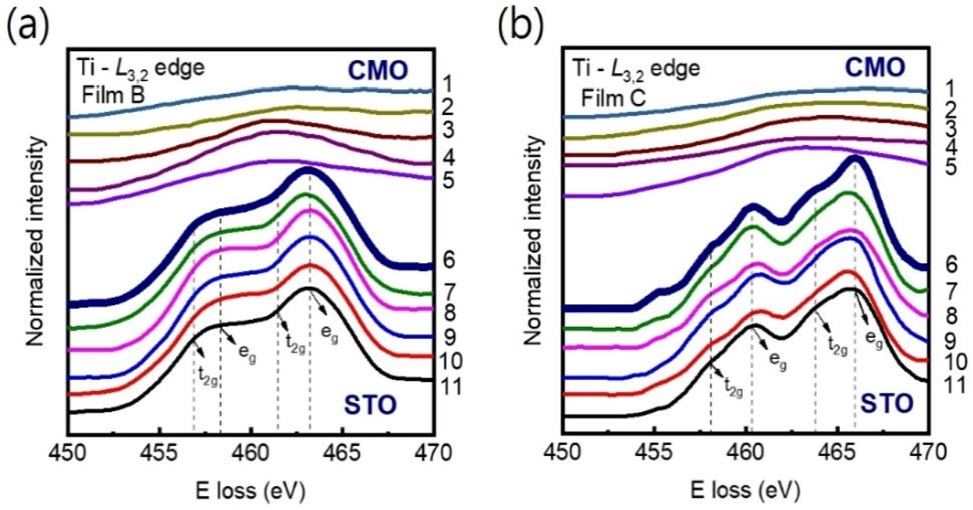
(a) and (b) EELS spectra at the Ti-L_3,2_ edge were measured across the film/substrate interfaces for films B and C, respectively at 11 different positions as indicated in Fig. 1. The peak positions of Ti-L_3,2_ remain unchanged from columns 6 to 11 for both films, indicating that the valence states of Ti remain constant on the STO side of the interface. The interface position (position 6) is marked by bold spectra. The spectra have been vertically displaced for clarity.

Our main finding is that strong tensile strain leads to a significant reduction in the Mn valence state, accompanied by an increase in oxygen vacancy concentration. This phenomenon is likely to result in an expansion of the unit-cell volume at the interface region.^28^ Note that, since the radius of Mn^4+^ ion (0.53 Å) is smaller than that of Ti^4+^ ion (0.60 Å), cationic interdiffusion at the interface also leads to a sharp increase in the out-of-plane-lattice constant. Nevertheless, this is not consistent with Ti-L_3,2_ spectra observed in Fig. 7(a) and (b). Energy-loss near-edge fine structures in the Ti-L_3,2_-edge can provide fundamental information on cation ordering and defect clustering such as oxygen vacancies.^31,48^ Based on the splitting of the 3d state into t_2g_ and e_g_ components, four main peaks can be seen in atomic-resolution EELS of Ti-L_3,2_ edges. Ti valence state decreases as the e_g_ peak positions shift to lower energies.^48^ However, the peak shift was not observed in Fig. 7(a) and (b), indicating almost no change in the Ti^4+^ valence state near the interface.

Furthermore, EDS measurements show no cation inter-diffusion across the film/substrate interface as discussed in the next. Notably, the Mn valence, not only at the interface but also in the entire sample of the CMO layer, is reduced compared to the value of the CMO bulk, implying the accumulation of oxygen vacancies throughout the entire CMO film. This is consistent with a slight expansion in the out-of-plane lattice constant in the bottom CMO layers, even away from the film/substrate interface, as observed in Fig. 6.

**Fig. 8:**
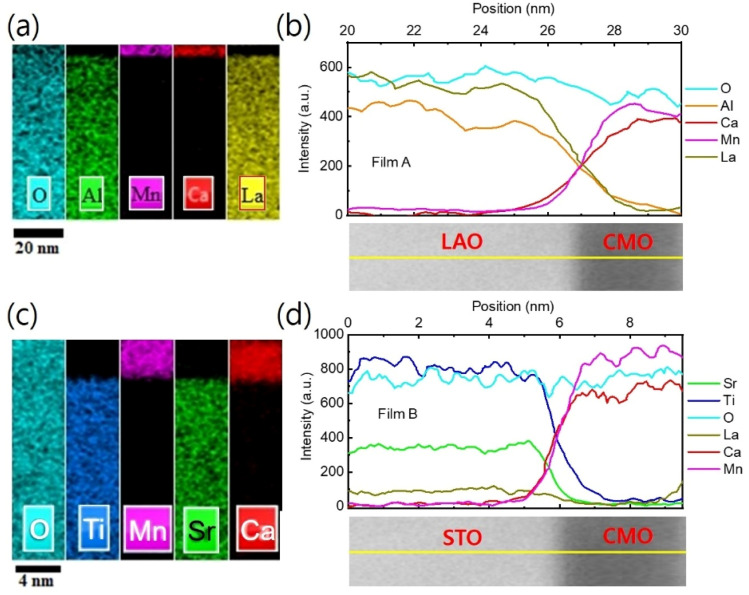
EDS mapping and line scan for film A [B]. (a) [(c)] EDS elemental mapping. (b) [(d)] EDS line scans plotted as a function of distance along the yellow line marked in the cross-sectional TEM images below.

EDS elemental mapping was used to study the chemical distribution around the film/substrate interface. Fig. 8(a)–(c) show the results for films A and B, with the elements represented in different colors. A uniform distribution of elements was observed in the films and substrates, and there was no appreciable interdiffusion of elements at the chemically distinct interface between the bottom CMO layer and substrate. The contribution of elements is more clearly seen in Fig. 8(b) and (d), where net counts are plotted as a function of distance, obtained by averaging the EDS results across the interfaces along the yellow marked lines of TEM images below. Chemical boundaries identified by the EDS signal correspond closely with sharp interfaces revealed by STEM, indicating no appreciable interdiffusion of atoms. However, direct observation of an increase in oxygen vacancy concentration at the interface region is not possible from Fig. 8 (a) and (d). Instead, the abrupt decrease in valence [Fig. 5(b) and (c)] and the small peak of tetragonality [Fig. 6(d)] at the interface serve as two smoking-gun fingerprints for the increase in oxygen vacancy concentration. In our previous study,^34^ we demonstrated that the abrupt decrease in valence is a result of electron loss from the oxygen atoms, which have converted into oxygen vacancies. The small peak of tetragonality is attributed to the crystal structure distortion around the oxygen vacancies.

Two potential causes of low Mn valence (Mn^2.3+^) at the strongly tensile strained interface are the electronic reconstruction induced by polar discontinuity and the accumulation of oxygen vacancies. Polar discontinuity originates from the mismatch between the polarities of the two materials at the interface, resulting in the formation of charged layers. These layers are subsequently screened by the accumulation of electrons, which may give rise to the emergence of low Mn valence states. This phenomenon has indeed been extensively observed in various oxide heterostructures, such as LaAlO_3_/SrTiO_3_ (ref. 21 and 49) and LaMnO_3_/SrTiO_3_.^50,51^ Conversely, the accumulation of oxygen deficiencies at the interface can also lead to the formation of low Mn valence states. Oxygen vacancies can function as electron donors, stabilizing the Mn^2.3+^ valence state. The latter scenario is considered less suitable, as electronic reconstruction typically occurs at interfaces with polarity discontinuity. However, no such polarity discontinuity exists at the interface between CMO and STO. Furthermore, we observed a structural change at the interface, accompanied by a valence change. This observation supports the scenario of oxygen accumulation as the primary cause. In light of these findings, the accumulation of oxygen vacancies emerges as the more plausible mechanism for the formation of low Mn valence states at the strongly tensile strained interface, as opposed to the electronic reconstruction induced by polar discontinuity. Further investigation is necessary to deepen our understanding of this phenomenon and its implications for the properties of oxide heterostructures. Note that structural changes may occur in a defective interface with a very low valence state. For example, the formation of a CaMnO_2.5_ brownmillerite-like structure (Mn valence of +3) might exist at the interface, where oxygen vacancies are ordered in a zig-zag arrangement, as reported in ref. 52. Nevertheless, there are other factors that prevent this change. Firstly, the oxygen-deficient interface with an ideal perovskite structure can be unstable or metastable, which means it could eventually transform into another structure in the bulk material. However, in our case, the oxygen-deficient structure is localized near the interface region. Even if the structure near the interface region is unstable or metastable, the overall stability of a given structure is determined by other parts of the sample that occupy the majority of the sample volume.

Finally, we will discuss the implications of the change in the Mn valence state in the film, particularly regarding its impact on the film/substrate interface. The two types of substrates result in distinct distributions of the corresponding Mn valence states at the interface. In the weakly tensile-strained film, the Mn valence of +3.5 is observed, while the strongly tensile-strained film exhibits an Mn valence of +2.3. Mn^3.5+^ represents an intermediate valence state of manganese. According to the bulk phase diagram, Mn^3.5+^ lies on the threshold of both ferromagnetic (FM) metallic and antiferromagnetic (AFM) insulating phases. As a result, the weakly tensile-strained heterointerface could, in principle, enable the switching on/off of the metallic/insulating nature using a weak perturbation, presenting valuable potential applications in electrical sensors. On the other hand, the Mn valence state of +2.3 suggests that the strongly tensile strained interface between the film and substrate is electron-doped. Electron-doped manganites based on CMO have been less frequently studied due to their complex phase diagrams. Despite this, these materials continue to attract attention in the research community. Several studies have investigated the properties of electron-doped manganites, such as Ca_1−*x*_Bi_*x*_MnO_3_,^53^ Ca_1−*x*_La_*x*_MnO_3−*δ*_,^54,55^ and Ca_1−*x*_Ce_*x*_MnO_3−*δ*_.^56,57^ Additionally, the reduction of the valence state can potentially explain the origin of the dead layer at the interface region. Several studies have provided evidence that the dead layer is not solely attributed to the thickness in manganate perovskite oxide heterostructures, but also to changes in interfacial strain,^58^ oxygen coordination,^6^ interfacial oxygen octahedral rotation,^7^ and concentration of oxygen vacancies.^8^ Therefore, the question of whether a dead layer exists in our samples poses an interesting topic that we will investigate in the future.

## Conclusions

Using spherical-aberration-corrected analytical scanning transmission electron microscopy and electron-energy-loss spectroscopy, we confirm the interplay between valence state, local structure, and oxygen vacancy near the film/substrate interface of the bottom CMO layer grown on different substrates. Our results show that the Mn valence state of the weakly tensile-strained film decreases monotonically from the CMO layer towards the film/substrate interface, while a region with an elongated out-of-plane lattice parameter and an abruptly lowered Mn valence state is observed near the interface of the strongly tensile-strained film. The significant reduction of the Mn valence state and the change in the local out-of-plane lattice constant for the strongly tensile-strained film are attributed to the presence of oxygen vacancies at the interface. These findings provide new insights into the control of interface properties through strain-driven thin-film technology.

## Author contributions

H.-J. Kim conceived the experiment's main idea. V.-H. Hoang prepared the samples, analysed the data, and wrote the original draft of the manuscript with assistance from H.-J. Kim. N.-S. Lee performed the STEM-EELS experiments. All authors contributed to comments and revision of the manuscript.

## Conflicts of interest

There are no conflicts to declare.

## Supplementary Material
